# Supplementary figures and images for: An untargeted exometabolomics approach to characterize dissolved organic matter in groundwater of the Samail Ophiolite
Source: Front Microbiol. 2023 Mar 9;14:1093372. doi: 10.3389/fmicb.2023.1093372 (PMC10033605; doi:10.3389/fmicb.2023.1093372)

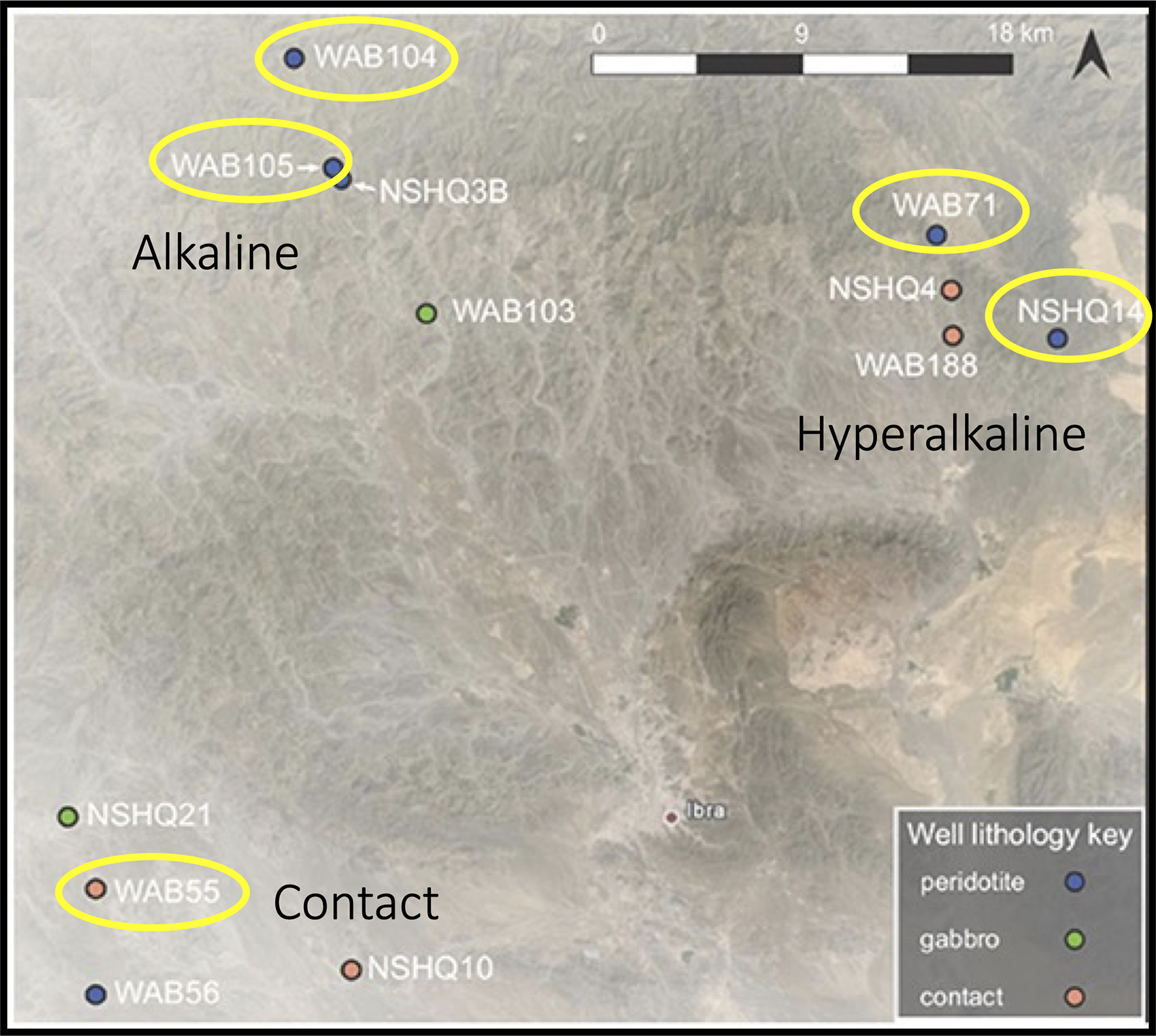

Supplement: Supplementary Figure 1 — Map of sampled wells in the Samail Ophiolite. Figure adapted from Rempfert et al. (2017). [file Image_1.TIFF]
